# Supplementary material for: Reassessing the modularity of gene co-expression networks using the Stochastic Block Model
Source: PLoS Comput Biol. 2024 Jul 29;20(7):e1012300. doi: 10.1371/journal.pcbi.1012300 (PMC11309492; doi:10.1371/journal.pcbi.1012300)
Supplement: Attachment — Submitted filename: SBM review response.pdf [file pcbi.1012300.s009.pdf]

Editor

All reviewers agree that there is utility in having models that can infer modularity in a network without imposing it a priori (for reasons the authors explain). It seems to me and the reviewers that the weighted Stochastic Block Model (SBM) is capable of identifying more modular clusters than other methods that use assortativity a priori. The two main issues that prevented us from being fully convinced are that some of us: 1) had difficulty understanding parts of the model, and 2) could not see whether the comparisons between SBM and other models (mainly WGCNA) were appropriate for distinguishing SBM as the preferred model. I shall discuss these two issues in turn.

Thank you for your time and the thoughtful comments on our manuscript. We hope the revised version of the manuscript addresses yours and the reviewers' concerns.

Description of the model both conceptually and mechanistically. Conceptually, I agree with Reviewer 1 that a better biological motivation is needed for introducing an assortativity-free model. We might easily imagine that some gene groups are not assortative, but real examples, and explanation or reasoning as to why they are not assortative, would go a long way to motivating the need for SBM (see Reviewer 1 paragraph on "A grounding in real biological examples").

This is a good point, and we now discuss other network organizations and provide examples of other types of network architecture for biological networks in the introduction (Lines 31-37 and Figure 1). However, to our knowledge, this issue has never been assessed in the context of gene co-expression networks, so we primarily discuss non-assortativity in gene co-expression in the discussion.

Mechanistically, Reviewer 1 and 2 had some uncertainty about the model and its implementation, which I was also unclear about. Specifically, a more detailed explanation of the description length, to better understand what it is calculating/estimating and what information it is capturing from the gene network.

We now provide a more complete and hopefully more intuitive description of the SBM in the methods and in the introduction (Lines 43-50 and 118-139).

Also, Reviewer 2 and 3 are uncertain about the choice of threshold for removing edges when the graph is reduced. What is considered a "viable set of genes" and why? Does removing these large p-value (with respect to correlation) edges have an effect on the results? Reviewer 2 suggests a small-scale study to elucidate these effects.

We provide a small set of simulations that show the behavior of FDR trimming (Fig 2), discuss the choice of FDR trimming more thoroughly in the methods (Lines 80-99) and in the discussion (Lines 388-402). We also provide a comparison of the clustering using the SBM in a smaller network with different FDR trimming in the SI (SI Fig 1). In short, the choice of FDR threshold does alter the density of the network, retaining only well-supported edges. This reduction in density leads to a smaller number of blocks and less subdivision of the blocks in lower levels of the hierarchy. However, the general pattern of the partition is robust to FDR choices, and the well-supported blocks are observed in most FDR levels.

Model comparisons. It is difficult to tell if the comparisons made are appropriate because different gene network clusters were compared without evidence that it would not effect the results. All reviewers rightly flagged this issue. Wouldn't it make more sense to use the same reduced gene network with all models?

This point is well taken, and we have now added an FDR trimmed version of WGCNA to SI (SI Fig 2) which allows for an exact comparison of the two models. This approach does not lead to significant changes in the WGCNA results (SI Fig 2). However, we think that it is important to compare each method using their respective, intended workflows. We couldn't find any application of WGCNA that uses p-value FDR correction; thus adding an FDR trimming step would be a novel and rather artificial application of their method—certainly unlike any other in the literature. Our approach to processing the gene expression correlations is an integral part of the clustering method we are comparing. Because our goal is to compare the full workflows, not the specific clustering on a particular network, we think that it is important to keep the main text results using WGCNA's proposed workflow.

And to include simulated gene networks (maybe several with distinct, but biologically realistic network topologies). to compare models? These comparisons using the same network for both (or all) models would be more convincing evidence if they showed that SBM captured something that WGCNA did not. Using more than one gene network type for comparison may also help readers understand the different instances where SBM excels or not compared to other models. Reviewer 1 gives more explanation on the use of simulated data, which I strongly suggest the authors consider

There are already several publications that that tackle the performance of SBM in simulated networks (see doi:10.1017/9781009118897 and references therein), and we have now highlighted that in the text (lines 230-233). We also acknowledge that it is important for the reader to get an intuitive feeling for this point without having to read such specialized literature; therefore, we have added a new main figure (3) illustrating through simple simulations the ability of the different methods to detect non-assortative modules. We do not, however, produce a full simulation study as it would be beyond the scope of this manuscript and largely redundant with the available literature.

Reviewer #1:

Understanding the modularity or integration of organisms is of fundamental importance in evolutionary genetics, at the level of phenotypes such as morphology and life-history and of gene expression (which can also be considered a phenotype). To understand these patterns on an organismal scale necessitates the use of big data such as gene expression data, but dealing with these data in a way that is interpretable presents a host of challenges. Often dimension reduction techniques must be used. Careful consideration of the assumptions in such models and how those assumptions can bias the biological interpretation of the results is necessary.

In this manuscript the authors explore the utility of using a weighted hierarchical stochastic block model to analyse networks of gene co-expression and identify biologically meaningful clusters of genes. Although there are several different methods that can be used to do this, most impose an a-priori modular structure on the network, which could bias the subsequent inference of the modularity of gene co-expression networks. The purported benefit of the SBM is that no structure or tuning parameters are imposed on the data, such that any type of network structure is possible. If this is the case, estimates of modularity or integration, and the biological interpretation of the blocks of co-expressed genes should be less biased.

The idea of this paper is great. However, the fundamental differences between this type of model and existing approaches were not explained well-enough to understand any costs of this approach. Although it imposes no structure on the data, presumably there are some features of the data, which are not necessarily biological, that would be more likely to lead to certain outcomes than others.

The introduction of the SBM was quite vague and generic. For example, the statement on line 36-40 that SBM can use any information contained in the gene co-expression network while other methods can't is unclear. All methods appear to be limited to the same information (the pairwise correlation between gene expression for every pair of genes in a network).

Thank you for the comments and kind words. To make the idea behind the SBM more clear and intuitive, we now provide a more complete and hopefully more intuitive description of the SBM in the methods and in the introduction (Lines 43-50 and 118-139).

It would be helpful to focus on introducing the conceptual differences between the SBM and other methods as they specifically relate to gene expression data, and any intricacies that go along with these types of data. A grounding in real biological examples would be useful as well. For example, Line 40- what other types of network organizations are expected in gene co-expression data?

We now add a figure (1) illustrating other possible network organizations in the introduction, for example, disassortative networks, and core-periphery networks (Lines 31-37).

A key aspect of this method that allows it to remain parameter free seems to be based on minimizing the description length. If this is the case, it would be very helpful if a more detailed explanation of the description length was given, and how it is estimated/calculated.

Thank you for raising this important point. While minimizing the description length is indeed a key aspect of the model, it is not the main feature that allows it to remain parameter-free. The non-parametric nature of the model primarily stems from the microcanonical formulation (e.g., assuming all its microstates are equally probable) and the careful choice of hierarchical priors.

In the microcanonical formulation, the model is defined in terms of hard constraints on the network structure, such as the exact degree sequence and the number of edges between blocks. This is in contrast to the more common canonical formulation, where these quantities are only fixed on average. Furthermore, the hierarchical priors used in the model are carefully chosen to be as uninformative as possible while still enabling the model to efficiently determine the optimal number of blocks to represent the network. These priors are defined over the model parameters, such as the edge counts between blocks, and are recursively integrated out of the likelihood function. This hierarchical structure allows the model to adapt its complexity to the data, without requiring any manual tuning of parameters.

The description length, which is equivalent to the negative log-likelihood of the model, can be seen as an alternative interpretation of the model that provides a more intuitive understanding of its behavior. Minimizing the description length is equivalent to finding the model that provides the most compact and efficient description of the data, balancing the trade-off between model complexity and goodness of fit. However, it is not the primary mechanism that makes the model parameter-free.

We have expanded the introduction and methods section to make these points clearer. (Lines 43-50, 118-158)

What features of the data other than real biological signal could affect the minimum description length or choice of model. For example, if correlations of larger magnitude disproportionately contribute, are those high correlations more likely to be estimated in more highly expressed genes because of higher power, are they more likely to be estimated with less error, etc?

It is indeed possible that such correlations could disproportionately contribute to the description length. However, this is not necessarily a problem, as one could argue that larger correlations are more likely to represent strong and more biologically meaningful relationships between genes. The model's ability to capture these strong correlations is a desirable feature, and we show that it allows for the identification of the most salient patterns in the data.

That being said, it is important to be aware of potential biases in the estimation of correlations, such as those arising from differences in gene expression levels or measurement error. These biases could potentially influence any model's performance and should be carefully considered when interpreting the results. One way to mitigate these biases is to use appropriate normalization and preprocessing techniques on the gene expression data before constructing the co-expression network. For example, we use Spearman correlations because they are substantially less sensitive to genes with large expression in some samples which can dramatically bias the correlation estimates.

A major drawback of this manuscript is that the method has not been validated with simulated gene expression data. The authors showed that the different methods give different answers (although also largely congruent answers), but which one is correct? Analysing simulated data would also allow the authors to explore how different features of the data (eg. Mean expression level, estimation error, etc) influence the results.

The performance of SBM in simulated networks has been addressed in previous publications (doi:10.1017/9781009118897, doi:10.1103/PhysRevResearch.2.043271). However, we agree with the reviewer; the reader should be able to get an intuition for the performance of SBM on simulated networks without having to read the highly technical studies. We have added a new main text figure (Fig 2) showing some simple simulations to illustrate the ability of the different methods in detecting non-assortative modules. These simulations demonstrate that SBM is capable of identifying both assortative and non-assortative clusters, as well as hierarchical structures, in the simulated networks. In contrast, methods like WGCNA, which rely on the assumption of assortative community structure, fail to capture non-assortative patterns in the data.

We also want to emphasize that producing a set of new simulated networks to compare the clustering algorithms is beyond the scope of this manuscript, and we believe that it will also be to a great extent redundant with the literature that has tackled this point using simulations (see refs cited above). Furthermore, the problem of accurately estimating gene co-expression networks from expression data is an open and active area of research, with many potential sources of bias and error that can influence the results. Fully addressing this issue would require a comprehensive investigation of different network inference methods, data preprocessing techniques, and simulation strategies, which again, we believe is beyond the scope of the current manuscript. Instead, we have chosen to focus on demonstrating the utility of our method for detecting biologically meaningful patterns in real gene expression data.

The authors compare the results of the proposed SBM method with one other method (WGCNA) using empirical data from D. melanogaster. They attempted to compare with a third method (MMC), but note that the third method performed poorly with their data, so it is not really discussed. The authors should either discuss why the third method performed poorly and treat it like a real comparison, or remove it from the manuscript altogether.

We have removed the MMC method from the manuscript.

Different edge weights were used for WGCNA and SBM which seems like a potentially important difference that could affect the results. For a true comparison the same gene co-expression network should be used for all methods even if it is possible to fit WGCNA and other methods with a denser network.

Please—see our answer to the editor’s comments above.

Overall, the manuscript is interesting and potentially important, but would benefit from 1) a clearer and more biologically grounded introduction; 2) validation of the approach using simulated data; 3) apples-to-apples comparison of the methods on empirical data.

We hope we successfully accomplished these improvements to the manuscript. Thank you again for your comments.

Reviewer #2

In this manuscript, Melo et al use the Weighted Stochastic Block Model (SBM) to cluster genes in expression data from Drosophila melanogaster. They compare results from SBM with results from the commonly-used WCGNA method and show that SBM identifies more modular clusters with less assortativity than WCGNA, instead capturing other notions of similarity. Overall the paper is clear and well-presented and the claims made are justified by the results. I have a small number of questions and comments I would like to see addressed in a revision.

- Speaking generally, I am convinced that going beyond assortativity is desirable, especially given the inherently circular logic used in such algorithms. However I don't end up with a great intuition for what exactly is driving the clustering if it isnt assortativity. This ends up coming through a bit in the methods and discussion eg "the amount of information required to encode the network". But this remains a bit abstract. It would be helpful to get some intuition for what this is capturing at the end of the introduction.

Thank you for your kind words. We now include a more thorough discussion of the idea and principles behind the SBM (Lines 43-50, 118-158), hopefully making the results more intuitive.

- On that note, if it is indeed harder to get an idea of what exactly relates the genes in the identified clusters, this may be a downside to the method that should be mentioned in the discussion.

We agree with the reviewer, it is difficult to get an intuition for what type of biological signal makes the genes 'stick together' in non-assortative clusters. We believe that part of the problem is that we have not been trained to understand or even imagine clusters that are not assortative/modular. We hope that this paper makes the community re-think modularity, the more non-assortative modules we find, the more intuitive it will be what types of patterns result in such clusters.

We now make it clearer in the text (Lines 45-48), genes are related not by the strength of their correlation to each other, but by the pattern of associations with every other gene. For example, Betzel et al. (doi:10.1038/s41467-017-02681-z) provides several examples of network organization besides assortativity, like disassortative networks, in which vertices have more edges to vertices between communities than within; or core-periphery, in which a central community is connected to other communities, but the peripheral communities are not internally connected. We now illustrate these possibilities in figure 1.

I am curious to hear more about the inclusion criteria for the genes and edges in the analysis:

- I'm not seeing a clear number of connections that can be included before you start running into computational limitations - eg the discussion around "We chose an FDR of 1% for the head and 0.1% for the body datasets which kept most of the genes while reducing the graph density to a manageable level for use in the SBM." This gives the number of genes but

presumably the runtime increases with the number of edges. How many edges are you including and what is the runtime? What does the scaling look like with the number of edges?

This is a good point; however, it is very difficult to assess because it depends strongly on the pattern of the network being analyzed and the shape of the posterior distributions (for example, if there is a single mode in the posterior distribution of blocks). While we could potentially compare full SBM fits at different FDR values, this would only be useful for our particular dataset and would demand several weeks of computational time. Our choice of network density was completely pragmatic, attempting to keep the run-time of the models under a week and still keep most of the expressed genes. To the first question, the head dataset (FDR 1%) and the body (FDR 0.1%) have around 500,000 edges, and the running time is approximately 1-2 weeks for the full workflow, this time varies due to the fitting being done using an MCMC chain. For a general discussion on the computational and algorithmic aspects of fitting the SBM, see doi:10.1103/PhysRevE.95.012317

- I am very curious about the information content of the removed edges, especially given you are using an inclusion criteria based on correlation, but a community detection method that is not. Is it possible to do a small-scale study on a smaller number of genes where you vary the FDR threshold eg from not removing anything down to a ~5% threshold? Would the major modules you find be the same? etc. If the communities detected are strongly influenced by this inclusion criteria, that would be a limitation of the analysis worthy of noting.

To address this issue, we now include a comparison of the SBM fit using a small subset of genes with a different FDR choices and show that the results are similar (SI Fig 1). Again, this is a good point, this is indeed a choice to be made for each study that can have consequences on the final clustering. Thankfully, in our workflow, it is basically the only free parameter, as we address in the discussion. Other methods have many more degrees of freedom. We now make this aspect of our workflow more explicit to the reader (Lines 96-99).

We highlight here and in the discussion (Lines 389-402) that it is expected that the results are not exactly the same given that the low-correlation high-p-value edges are extremely noisy. Therefore, all methods address this issue in a way or another. WGCNA deals with this by transforming edges with small correlations to zero via an exponent (soft thresholding), while we prune them using an FDR threshold.

Digging deeper into the information content lost by removing edges is a very interesting question, but we believe it will be productive only in the hypothetical scenario where a study has sample sizes high enough to estimate ALL correlations properly; however, these are far and few (and any actually exist). For limited-sample size studies, the alternative, of adding poorly estimated correlations, is not productive. SI Fig 1 shows that the main structure of the clustering is robust to different FDR choices.

- I am also curious about the decision to correct for unknown covariates. Of course, this is a standard preprocessing step, but the way these methods typically work would suggest they may be likely to remove some large network structure prior to network analysis. I realize there isn't really a “right” choice to be made here, but I am curious if the authors considered what the results would look like without this correction. For example, would some of the

identified modules lack biological interpretability and thus be more likely to represent unobserved covariates?

This is a very important point: what is the effect of data pre-processing on downstream analysis? It is out of the scope of this paper to do a thorough exploration of the effect that every pre-processing step will have on the clustering approach, but we acknowledge the importance that pre-processing quality control (QC) steps have, not only for our analysis but for any analysis of gene expression data.

In general, we don't use unknown covariate discovery and removal in our analyses unless totally warranted. However, in this particular dataset, a very obvious structural pattern remained after accounting for known experimental covariates (see below the example for head transcriptomes). Besides the known covariates (colored in the plots below), an exploration of the (lack of) genetic structure in our population also indicated that such batches didn't have an obvious biological explanation. Without correcting for these unknown covariates, we would be interpreting experimental noise as biological signal, severely biasing the correlation estimates and introducing non-biological patterns into the covariance structure.

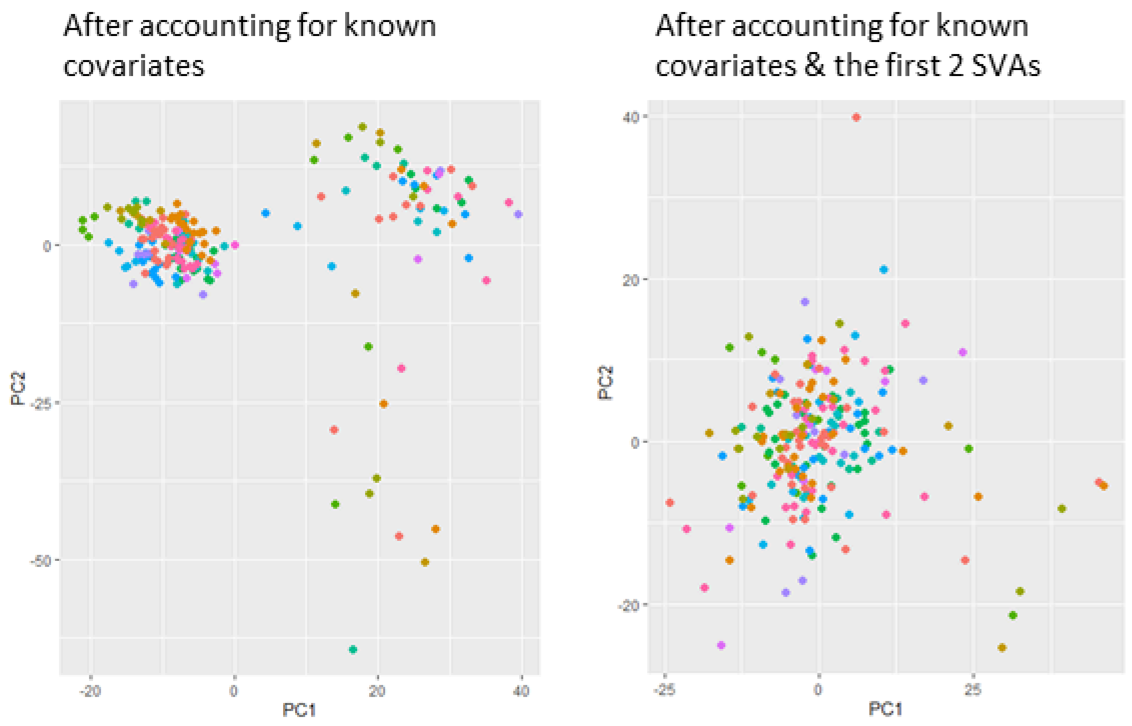

Ideally, the known batches would lead to well-behaved gene expression distributions, but this is rarely the case in RNAseq experiments, and other data cleaning procedures are necessary. These artificial patterns are clearly visible in the PCA plots, where distinct clusters of samples can be observed that do not correspond to any known biological factors. These clusters likely represent technical artifacts or batch effects that were not accounted for by the known covariates. Failing to remove these unknown covariates would result in the identification of modules that are driven by technical factors rather than true biological relationships.

By correcting for unknown covariates using surrogate variable analysis (SVA), we aim to mitigate the impact of these confounding factors and ensure that the identified modules reflect genuine biological patterns. This correction helps to improve the biological interpretability of the results and reduces the risk of drawing false conclusions based on technical artifacts.

We acknowledge that the choice of whether to correct for unknown covariates is not always straightforward and depends on the specific characteristics of the dataset and the research objectives. However, in our case, the presence of clearly artificial patterns in the uncorrected data strongly supports the need for this preprocessing step to obtain biologically meaningful results.

Reviewer #3

The manuscript introduces an important discussion about the nature of network communities/clusters/blocks in gene networks. Specifically that there may be important clusters that do not follow the commonly held assumption of being assortative and that adhering to strongly to this assumption may negatively restrict our findings when analysing such networks.

I think this is an important argument to make, but there are technical issues with the current version of the manuscript that risk to undermine this message.

Thank you for the kind words, we hope to address these issues below.

1. The whole premise is based on a comparison between modular methods on one network and the SBM on a different network. This does not really create a fair comparison since both the network and the method change. One solution might be to add another method: apply the Louvain method (or alternative modularity maximization method) to the same network as used for the SBM.

See answer to the editor above. Also, please note that modularity maximization is inherently problematic and has been extensively compared to the SBM, with a clear advantage for the SBM in inferential contexts (doi:10.1103/physreve.108.024309). Thank you for pointing this out, we needed to highlight this point, and edited the main text accordingly (see lines 231-234).

2. Thresholded correlation matrices do not make good network representations. The authors acknowledge this in the discussion and point to more principled methods of reconstruction, so why not use them to construct the network? I understand that the approach used by the authors may be standard practice, but it is relatively straightforward to apply the graphical LASSO to a covariance matrix using available software (e.g., in python/R).

We first note that we are not using the usual thresholding on absolute correlation value, but instead attempting to keep edges for which we have good statistical support, even if they are weak. We now make this clearer in the methods and discussion (Lines 79-99 and 396-403). Fig 3 now also shows how this method performs in a simple known correlation matrix, and SI fig 1 shows the manageable impact that different FDR choices have on the results.

Regarding the graphical LASSO and other such methods, they are fundamentally attempting to answer a different question. In working with gene co-expression networks, and not with gene regulatory networks, we are not interested in the partial associations between genes, which are more indicative of regulation relations. Instead, we are interested in associations (not necessarily causal) that may inform function. This may be a subtle point, but it is crucial to understand the difference between the two approaches. In an evolutionary context, what matters is precisely the correlations, not the causal effects, because it is the correlations that encode the evolutionary constraints and underlying genetic architecture responsible for the pattern of gene expression. Our results show that genes that are functionally related tend to

have similar patterns of co-expression, even if they do not directly regulate each other. This co-expression can arise due to shared regulatory mechanisms, common evolutionary history, or the need for coordinated expression to carry out specific biological processes. Therefore, the correlation structure of gene expression, rather than the causal relationships driving the correlation between genes, is what captures the evolutionary constraints and functional relationships that shape the organism's phenotype that we are interested in.

3. The block-wise measure of assortativity is presented poorly. (i) assortativity and modularity are related, but distinct statistics (see Newman 2003). (ii) The presented min and max values are wrong: +1 is not possible and the minimum is -0.5 (e.g. bipartite graph) (min and max values of assortativity are not trivial, see Cinelli et al 2020). (iii) The values of the block assortativity proposed depend on the assortativity of the other blocks, so the values might not have the interpretation the authors think they have. There are other methods for examining the assortativity at finer resolutions than the whole network (e.g., Peel et al 2018 + Cantwell and Newman 2019) It might be better to use the ratio of in/out edges or a heatmap of the (normalised) e_rs - e_r^2 matrix (or both).

Regarding point 1, we are not sure if this is correct: Newman's (categorical) assortativity r is exactly the same as the modularity (Q) we have on page 8 except for the normalization ($Q \in [-1/2, 1]$ and $r \in [-1, 1]$). Unlike r, the maximum and minimum values of Q are easier to obtain, precisely because of the normalization: B perfectly assortative communities result in a trivial modularity of $Q = 1-1/B$, which approaches arbitrarily close to 1 with $B >> 1$ (therefore, we would not call this impossible). However, it is true that the possible values of Q in practice can depend on other aspects of the network, which we now make clear (Lines 204-210). This makes comparison of modularity values across networks in different organisms or using a different number of genes difficult, but this can still be a useful metric across similar tissues, like we do here.

As for point 2, the range of [-1/2, 1] applies to the global modularity, not the local assortativity values q_r. This comment made us return to Peixoto (2018), which is where we got $q_r \in [-1, 1]$. This is indeed incorrect, although it is difficult to observe values outside this range. The true range appears to be $q_r \in [-B, B/2]$ (if $B > 1$) for each individual r. This would happen if, for example, we had $e_{rr} = E$ for a particular group r (that is, half of all edges connect nodes from the same group), and the rest was distributed arbitrarily among the other B-1 groups. In this case, we would have $q_r = B/2 \geq 1$ for this r, which implies $q_s < 1$ values for other groups. Therefore, values outside [-1, 1] can occur if the groups have quite unequal degree sums. Having said this, the normalization of the q_r are not that important for their interpretation. If Q is meaningfully estimated, q_r measures the contribution of each group, with the caveats mentioned above. The most important aspect, and the aspect we highlight, is the sign of each q_r, which is related to assortativity/disortativity. The alternatives proposed here are not much better, for example: $e_{in}/e_{out} = e_{rr}/(e_r - e_{rr})$ is not as clear because we can have values that are > 1 and still contribute positively to modularity.

Some minor points:

- Clustering all genes could be considered a negative as much as a positive given that most clustering algorithms will assign all input entities to a cluster.

This is specially relevant in comparison to WGCNA, that has a "non-clustered" category that can be sizeable in practice, only clustering 10% of the genes (for example, see doi:10.1101/gr.257592.119). Also, the SBM is unique in that it will avoid overfitting even if this means placing all vertices in the same cluster, unlike modularity maximization (doi:10.1103/PhysRevResearch.2.043271).

- "Level-1 block 12 can also be referred to as 12-7-2-2-1 (see fig. 1" -- this does not correspond to anything in fig 1

Fig 1 is a schematic that informs how to read all the labels, not this one in particular. We have altered the text to make this clear (Lines 282-283).

- The parameters used to threshold the correlation matrix seem arbitrary. It would be good to have some justification and/or robustness check.

Please—see answer above to the editor and reviewer 2.

Cantwell, G. T., & Newman, M. E. J. (2019). Mixing patterns and individual differences in networks. _Physical Review E_, _99_(4), 042306.
Cinelli, M., Peel, L., Iovanella, A., & Delvenne, J. C. (2020). Network constraints on the mixing patterns of binary node metadata. _Physical Review E_, _102_(6), 062310.
Newman, M. E. (2003). Mixing patterns in networks. _Physical review E_, _67_(2), 026126.
Peel, L., Delvenne, J. C., & Lambiotte, R. (2018). Multiscale mixing patterns in networks. _Proceedings of the National Academy of Sciences_, _115_(16), 4057-4062.
