# Supplementary material for: Reassessing the modularity of gene co-expression networks using the Stochastic Block Model
Source: PLoS Comput Biol. 2024 Jul 29;20(7):e1012300. doi: 10.1371/journal.pcbi.1012300 (PMC11309492; doi:10.1371/journal.pcbi.1012300)
Supplement: Attachment — Submitted filename: Review_Zambelli.docx [file pcbi.1012300.s010.docx]

The authors present the outcomes of different community partition algorithms, specifically WGCNA and Weighted-Nested Degree Corrected SBM. They highlight the ability of the latter to generalize community recognition based not only on assortativity but also on similarity in connectivity patterns. This work is particularly interesting as it emphasizes that statistically and biologically significant blocks are not necessarily highly assortative, challenging a common assumption in such analyses.

Overall, the article is clear and easily readable, effectively conveying a key message for the topic in a simple and direct manner.

A minor consideration: The section “Weighted SBM” (Line 159) lacks clarity in some parts, likely due to some typos. In particular, Line 163 could benefit from a clearer distinction between the weight transformation process and the statistical model chosen as the likelihood for the edge weights.

Additionally, did you repeat the SBM fitting procedure to check if the community partition outcomes remain consistent given the algorithm's intrinsic stochastic nature?
